# Bacterial contamination of healthcare workers’ attire

**DOI:** 10.1017/ash.2025.10218

**Published:** 2025-12-10

**Authors:** Lauro Damonti, Mariana Marti, Sara Droz, Luca Di Caprio, Jonas Marschall, Philipp Jent

**Affiliations:** 1 Department of Infectious Diseases, Inselspital, Bern University Hospital, University of Bern, Bern, Switzerland; 2 Institute for Infectious Diseases, University of Bern, Bern, Switzerland; 3 University of Arizona College of Medicine - Phoenix, Phoenix, AZ, USA

## Abstract

Healthcare workers’ attire is prone to microbial contamination, potentially facilitating pathogen transmission. In our study, attire was almost universally contaminated, mainly with skin commensals; among non-commensals, *Staphylococcus aureus* predominates. Sleeves were less contaminated, possibly due to “bare below the elbows” policies. Easier access to dispensers may increase attire change.

## Background

Contamination of healthcare workers (HCWs) attire occurs quickly, often within hours, and increases with duration of use; daily attire changes are recommended to limit contamination risk.^1,2^ However, the role of attire contamination in the horizontal transmission of multidrug-resistant organisms (MDROs) and healthcare-associated infections (HAIs) remains unclear, as most studies demonstrate have colonization rather than actual transmission.^3–6^ The aim of this study was to investigate bacterial colonization of HCWs’ attire used in the clinical setting at a tertiary university hospital and to identify factors influencing attire exchange as well as those associated with colonization by MDROs.

## Methods

**Figure 1.**
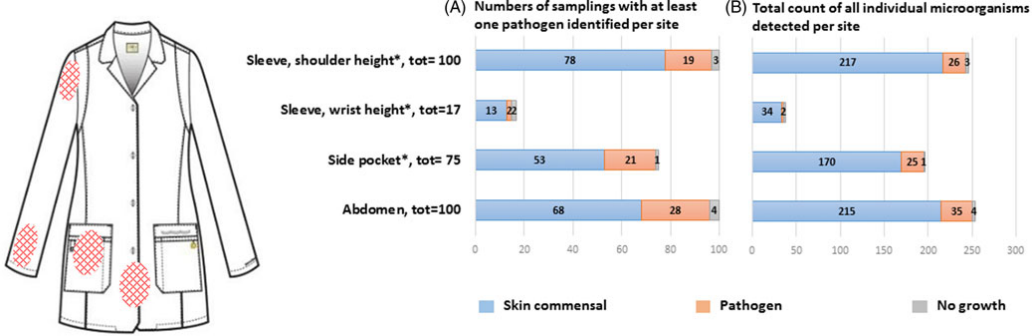
A. Representation of the number of samplings with evidence of growth of at least one pathogen at the four sites B. Total count of all individual microorganisms detected per site (a single site can show growth of more than one pathogen and skin  commensal). At the site “sleeve, wrist height”, no growth was detected in 2 samples (not shown on the graph for the sake of compression). The hatched area indicates the sampled zone. The asterisk (*) denotes the dominant side.

Over five afternoons in May and June 2022, one hundred consecutively selected voluntary HCWs were interviewed, and microbiological samples of their used attire were collected upon return to the automated clothing delivery and disposal system. The interview consisted of a standardized electronic survey on a laptop provided on-site; the collected data were automatically transmitted to a dedicated Research Electronic Data Capture (REDCap) database. The survey contained details on use of the respective work attire, clinical department, and distance from their workplace to the clothing delivery system. In parallel, a physician and a nurse from the infection prevention and control unit performed standardized microbiological sampling on the outermost layer of each used attire set using Eswab® Collection kit Regular (Becton Dickinson, Eysins, Switzerland) at a maximum of four sampling sites across four types of attire, all made of cotton including scrubs, pocketed coats, and two types of pocketless polo shirt (Figure 1). Swabs were applied in a Z-shaped line once horizontally and once vertically within a 10 x 10 cm area, which was delimited using a template for standardization. Samples were cultured on selective plates, incubated for 48 hours at 35 °C, with CO_2_ supplementation when required. Bacterial growth was semi-quantitatively scored from 1 to 4 crosses following standard laboratory protocols, identified by MALDI-TOF, and tested for antibiotic susceptibility using the Kirby-Bauer method according to EUCAST criteria. Possible skin contaminants were identified according to guidance from the Centers for Disease Control and Prevention (CDC) National Healthcare Safety Network (NHSN) Patient Safety Component Manual.^7^ Fungi were excluded from the analysis. At Bern University Hospital, the laundry exchange process is fully automated: HCWs can retrieve new clothing and return used attire at a dedicated automatic delivery system, which are subsequently laundered by an external company using a standardized process (60 °C for 50 mins). The formal recommendation at our institution is to change work clothing daily, or immediately if visibly soiled, but no monitoring of compliance is performed. Unlike practices in some other countries, wearing or washing work attire outside the premises is not permitted. Our primary objective was to perform a descriptive analysis of the participants and to illustrate the distribution of bacterial contamination at different locations on HCW attire. Our secondary objective was to use uni- and multivariate logistic regression models to identify factors associated with the frequency of attire changes (end point: changing attire on the same day) and contamination with clinically relevant pathogens. Variables were included via forward selection and then backward deletion, with *p* < 0.1 as the inclusion criterion. Variables were checked for collinearity before inclusion in the model. All analyses were performed with R (version 4.0.2). A *P* value < 0.05 was considered statistically significant.

## Results

Baseline characteristics of the hundred participants are summarized in Table 1. Most of them were women (77%) and nurses (45%). The vast majority (90%) had interaction with patients (inpatients, outpatients, or both), yet encounters with patients placed under contact precautions were less commonly reported (17%). 81% required less than five minutes to access the automatic delivery system, while the median time the analyzed set of attire had been worn was 9 hours [2–100h].

**Table 1.** Baseline characteristics of the participants

Characteristics	Nurse, *N* = 45^1^	Physician, *N* = 16^1^	Other, *N* = 29^1^	No contact, *N* = 10^1^
Female gender	41 (91%)	6 (38%)	23 (79%)	7 (70%)
Age	29 (25, 38)	32 (26, 35)	29 (27, 54)	48 (32, 54)
**Type of patient interaction**				
Inpatients	25 (56%)	9 (56%)	6 (21%)	0 (0%)
Outpatients	6 (13%)	2 (12%)	8 (28%)	0 (0%)
In- and outpatients	14 (31%)	5 (31%)	15 (52%)	0 (0%)
Patients under contact precautions	11 (24%)	2 (12%)	4 (14%)	0 (0%)
No interaction with patients	0 (0%)	0 (0%)	0 (0%)	10 (100%)
**Type of clothes**				
Coat	1 (2.2%)	10 (62%)	3 (10%)	4 (40%)
Polo	5 (11%)	0 (0%)	15 (52%)	3 (30%)
Scrub	39 (87%)	6 (38%)	11 (38%)	3 (30%)
**Building**				
INO^2^	29 (64%)	3 (19%)	17 (59%)	5 (50%)
Main	15 (33%)	8 (50%)	3 (10%)	0 (0%)
Other	1 (2.2%)	5 (31%)	9 (31%)	5 (50%)
**Time to the automatic delivery system**				
< 5 min	37 (82%)	11 (69%)	24 (83%)	9 (90%)
> 5 min	8 (18%)	5 (31%)	5 (17%)	1 (10%)
**Clothing wearing time**				
> 1 day	4 (8.9%)	7 (44%)	6 (21%)	5 (50%)
Same day	41 (91%)	9 (56%)	23 (79%)	5 (50%)

^1^ *n* (%); median (IQR).

^2^Interdisciplinary building with Intensive Care Units, Emergency Department, and Operation Theaters.

Results of sampling are shown in Figure 1. Each of the four sites was sampled when feasible, depending on clothing type and the availability of the areas to be tested. The vast majority of samples (97%) exhibited microbiological growth by semi-quantitative culture, primarily attributed to common skin commensals. Evidence of growth of at least one pathogen (ie, non-commensal organism, Figure 1A) was found in 28 of 100 abdominal samples (28%) and 19 of 100 shoulder-level sleeve samples (19%). Among 75 side-pocket samples, 21 were positive for a pathogen (28%), while 2 of 17 wrist-level sleeve samples tested positive (12%). Similar findings were observed when the total count of all individual microorganisms was considered (Figure 1B). *S. aureus* was the most frequently identified pathogen at each localization, varying from 6% at the wrist site to 17% at the pocket location. No methicillin-resistant *S. aureus* was found. Enterobacterales were identified on 11% of the attire, and non-fermenting bacteria were detected at the same rate. Susceptibility testing was conducted for all samples, revealing three pathogens (*Rahnella aquatilis, Pantoea septica,* and *Pseudomonas massiliensis*) with varying degrees of resistance. In an additional analysis, we examined factors influencing the frequency of HCW attire changes, finding an association with shorter walking times to the automated laundry dispenser (< 5 mins vs longer) and a possible, albeit marginal, association with younger age and being a nurse. Due to the limited number of pathogen-colonized attire sets, we were not able to find any predictors for contamination with relevant bacterial pathogens.

## Discussion

Our data highlight the significant and extensive contamination of medical attire used by HCWs from various locations, even after a single day of use, which is consistent with previous reports. However, most of the detected bacteria were common skin commensals, while only a fraction of the positive cultures revealed potential pathogens, such as *Staphylococcus aureus*. In a setting characterized by low MDRO prevalence, we detected no MDROs on HCW attire in our study. Surprisingly, sleeves did not exhibit higher contamination levels compared to other areas, which may reflect both the limited use of long sleeves due to the widely adopted “bare below the elbows” policy^2^ and good hand hygiene practices (80% on average, based on 2 310 observations at our institution in 2024), reducing the actual deposition of pathogens at wrist height. Our in-depth analysis suggests that proximity to the automatic delivery system is associated with a higher frequency of changes. Our study is limited by the small number of observations, the short time frame (May–June) which does not account for seasonality, and sampling restricted to the upper body. We could not determine the optimal duration of attire use or the role of attire colonization in organism transmission and we were unable to assess whether certain attire types or professional groups were associated with higher contamination. Finally, our findings only may be generalizable to settings with a low prevalence of MDROs.

## Conclusions

HCW attire appears to become contaminated with as little as one shift, but mainly by skin commensals. Non-skin-commensal bacteria or MDROs were rarely detected in our setting, and most commonly in side pockets or the abdominal area of attire. Easier access to automated systems could boost the frequency of attire changes. Further studies to assess the impact on HAI and MDRO transmissions are warranted.

## Supporting information

10.1017/ash.2025.10218.sm001 Damonti et al. supplementary material Damonti et al. supplementary material

## Data Availability

All data can be made available upon request to the corresponding author.

## Supplementary material

The supplementary material for this article can be found at https://doi.org/10.1017/ash.2025.10218.
