# Supplementary material for: Bacterial contamination of healthcare workers’ attire
Source: Antimicrob Steward Healthc Epidemiol. 2025 Dec 10;5(1):e336. doi: 10.1017/ash.2025.10218 (PMC12722543; doi:10.1017/ash.2025.10218)
Supplement: Damonti et al. supplementary material [file S2732494X25102180sup001.docx]

SM Table 1: Estimated predictors for frequency of changing clothing from the uni- and multivariable logistic regression model

|  | Univariable analyisis | | | | Multivariable analysis* | | | |
| --- | --- | --- | --- | --- | --- | --- | --- | --- |
| Endpoint = change the same day (0/1) | **Odds ratio** | **95% CI** | | **p-value** | **Adjusted**  **Odds ratio** | **95% CI** | | **p-value** |
| Time to the automatic delivery system |  |  |  |  |  |  |  |  |
| < 5 minutes | Ref. |  |  |  |  |  |  |  |
| > 5minutes | 0.21 | 0.07 | 0.62 | <0.001 | 0.12 | 0.03 | 0.48 | 0.003 |
| Male gender | Ref. |  |  |  |  |  |  |  |
| Female | 1.81 | 0.63 | 5.18 | 0.27 |  |  |  |  |
| Age group (< 30y) | Ref. |  |  |  |  |  |  |  |
| > 30y | 0.28 | 0.09 | 0.83 | 0.02 | 0.24 | 0.06 | 0.92 | 0.04 |
| Profession |  |  |  |  |  |  |  |  |
| nurse | Ref. |  |  |  |  |  |  |  |
| physician | 0.13 | 0.03 | 0.52 | <0.001 | 0.16 | 0.03 | 0.74 | 0.02 |
| other | 0.37 | 0.10 | 1.46 | 0.16 | 0.37 | 0.09 | 1.62 | 0.2 |
| no contact | 0.10 | 0.02 | 0.49 | <0.001 | 0.11 | 0.02 | 0.62 | 0.02 |
| Type of clothes* |  |  |  |  |  |  |  |  |
| coat | Ref. |  |  |  |  |  |  |  |
| polo | 9.36 | 2.24 | 39.12 | <0.001 |  |  |  |  |
| scrub | 35.75 | 8.41 | 151.96 | <0.001 |  |  |  |  |
| Building* |  |  |  |  |  |  |  |  |
| INO | Ref. |  |  |  |  |  |  |  |
| Main | 2.40 | 0.48 | 12.01 | 0.29 |  |  |  |  |
| other | 0.16 | 0.05 | 0.51 | <0.001 |  |  |  |  |

*variables were included via forwards selection then backwards deletion with p<0.1 as inclusion criteria. However, the variables “type of clothes” and “building” were not included in the analysis, due to collinearity with “profession” and time to “time to the automatic delivery system”, respectively.
